# Snap-jaw morphology is specialized for high-speed power amplification in the Dracula ant, *Mystrium camillae*

**DOI:** 10.1098/rsos.181447

**Published:** 2018-12-12

**Authors:** Fredrick J. Larabee, Adrian A. Smith, Andrew V. Suarez

**Affiliations:** 1 Department of Entomology, National Museum of Natural History, Smithsonian Institution, Washington, DC, USA; 2 Department of Entomology, University of Illinois, Urbana-Champaign, Urbana, IL, USA; 3 Department of Animal Biology, University of Illinois, Urbana-Champaign, Urbana, IL, USA; 4 Beckman Institute for Science and Technology, University of Illinois, Urbana-Champaign, Urbana, IL, USA; 5 Research and Collections, North Carolina Museum of Natural Sciences, Raleigh, NC, USA; 6 Department of Biological Sciences, North Carolina State University, Raleigh, NC, USA

**Keywords:** finite-element analysis, power amplification, ants, microCT, functional morphology

## Abstract

What is the limit of animal speed and what mechanisms produce the fastest movements? More than natural history trivia, the answer provides key insight into the form–function relationship of musculoskeletal movement and can determine the outcome of predator–prey interactions. The fastest known animal movements belong to arthropods, including trap-jaw ants, mantis shrimp and froghoppers, that have incorporated latches and springs into their appendage systems to overcome the limits of muscle power. In contrast to these examples of power amplification, where separate structures act as latch and spring to accelerate an appendage, some animals use a ‘snap-jaw’ mechanism that incorporates the latch and spring on the accelerating appendage itself. We examined the kinematics and functional morphology of the Dracula ant, *Mystrium camillae*, who use a snap-jaw mechanism to quickly slide their mandibles across each other similar to a finger snap. Kinematic analysis of high-speed video revealed that snap-jaw ant mandibles complete their strike in as little as 23 µsec and reach peak velocities of 90 m s^−1^, making them the fastest known animal appendage. Finite-element analysis demonstrated that snap-jaw mandibles were less stiff than biting non-power-amplified mandibles, consistent with their use as a flexible spring. These results extend our understanding of animal speed and demonstrate how small changes in morphology can result in dramatic differences in performance.

## 1. Introduction

Animal speed is a key performance trait that can determine the outcome of many ecological interactions, such as those between predator and prey. Mechanistically, speed reflects the fundamental relationship between form and function of musculoskeletal systems. Movement speed is determined, in part, by muscle power which is a trade-off between muscle force and velocity [1]. Many animals have overcome the innate power limits of muscle by incorporating latches and springs to reduce the time over which a muscle works. For example, the fastest movements are found among the defensive and prey capture behaviours of arthropods like trap-jaw ants, mantis shrimp and froghoppers [2–5]. In these groups, slow muscles store potential energy in an elastically deformable spring, then release it with a fast-acting latch release mechanism. The performance output of these power-amplified movements is determined by complex interactions among the components' morphology and physiology [6], making them powerful systems for studying form–function relationships. Here we seek to better understand the limits of arthropod appendage performance and how morphology is specialized for power amplification by examining a novel snap-jaw mechanism in the ant genus *Mystrium*.

In contrast to other power-amplified movements, where separate structures act as latch and spring to accelerate an appendage, some insects use a ‘snap-jaw’ mechanism that incorporates the latch and spring on the accelerating mandible itself. They press the tips of their mandibles together to build potential energy that is released when one mandible slides across the other, similar to a human finger snap [7,8]. Snap jaws are fundamentally different from other power-amplified appendages, and have evolved in two genera of ants and at least two genera of termites [8–11]. However, detailed characterization of snap-jaw mechanisms are still lacking, representing a significant gap in our knowledge of animal performance.

**Figure 1.**
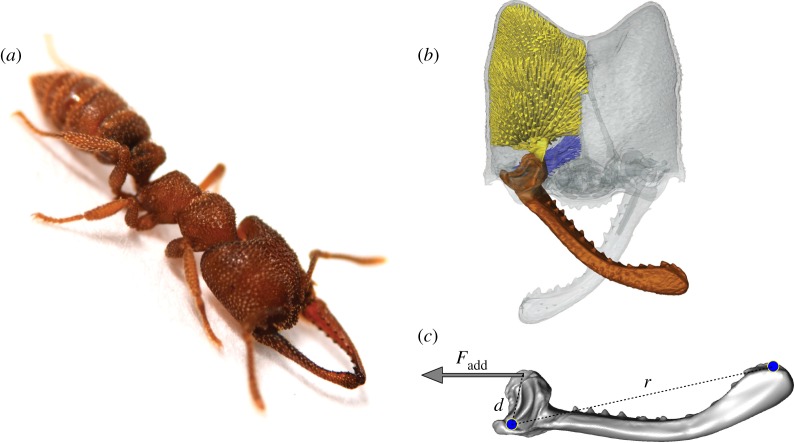
Morphology of the snap-jaw Dracula ant, *Mystrium camillae*. (*a*) Still image of a major worker with mandible tips touching in preparation for a strike. (*b*) Three-dimensional surface rendering of the head (grey), mandible (brown), adductor muscle (yellow), and abductor muscle (blue) involved in mandible movement. (*c*) Surface model of the mandible displaying some measurements for kinematic analysis and parameters for finite-element analysis. Blue dots represent points of finite-element model constraint (ventral condyle not visible). *d*, in-lever length between mandible centre of rotation and point of muscle attachment; *F*_add_, force applied to mandible by mandible adductor muscle; *r*, mandible length.

In ants, snap jaws are best known from the genus *Mystrium* (subfamily Amblyoponinae) (figure 1), often called Dracula ants because of their non-destructive feeding on larval haemolymph [12,13]. They are known to use their snap jaws for predating on leaf litter arthropods and also probably for defence [11,14,15]. They are one of at least six lineages of ants that have evolved power-amplified mandibles [16], providing an opportunity to examine convergent evolution and how the morphology of specific components are adapted for high-speed movements. In addition, many species of *Mystrium* have distinct morphological castes that differ in body and head size [17], allowing for comparisons of performance and morphology within species.

To better understand the morphological features that facilitate power-amplification mechanisms, generally, and the snap-jaw mechanism, specifically, we examined the performance and morphology of the Dracula ant, *Mystrium camillae*. We used high-speed videography to estimate the kinematics of *M. camillae* mandible snaps and confirm that they are power amplified. We also used finite-element analysis to simulate how *M. camillae* mandibles of different castes deform in response to muscle forces mimicking the loading phase of a strike. Finally, we compared mandible morphology of *Mystrium* to their non-power-amplified relative *Stigmatomma* to test the hypothesis that *Mystrium* mandible morphology is specialized for power amplification.

## 2. Methods

### 2.1. Study organisms

The genus *Mystrium* (subfamily Amblyoponinae) is restricted to tropical Africa, Australia and Southeast Asia [17,18]. *Mystrium camillae*, the species we examined, nest and forage underground and in the leaf litter, and because of their cryptic habits, are relatively infrequently collected [11]. A colony of *Mystrium camillae* was collected in Maliau Basin Conservation Area in Sabah, Malaysia in August 2014.

A colony of *Stigmatomma pallipes* (also subfamily Amblyoponinae) consisting of approximately 20 individuals and 8–10 larvae was collected from Duke Forest in Durham, NC, USA in August 2017. Despite being closely related and having similar mandible morphology to *Mystrium* [19], *Stigmatomma* jaws are not power amplified (see electronic supplementary material), making them an excellent system for identifying morphological features required for the evolution of ultrafast jaws.

All ants were kept in the laboratory in artificial nests constructed from plastic boxes coated in Fluon (Northern Products), and a plaster-filled Petri dish. The ants were provided water and sugar daily, fed live crickets or termites three times a week, and kept at 25°C and a 12 h light-dark cycle.

### 2.2. Kinematic analysis

The general methods for high-speed recording of mandible strikes were similar to those used in a previous study [20]. Ants were restrained to an insect pin on the dorsal surface of the head with dental wax and fitted onto a micromanipulator. Strikes were elicited with gentle puffs of air or by touching the lateral surface of the mandible with an insect pin. The loading phase prior to a strike was recorded with Phantom Miro eX4 high speed camera (Vision Research Inc., Wayne, NJ) filming 1000–6000 frames s^−1^ and shutter speeds of 6000 s^−1^ through a 105 mm F/2.8 1 : 1 macro lens (Sigma Corporation of America). To resolve complete mandible movements, strikes were filmed with a Photron Fastcam SA-X2 (Photron USA, Inc., San Diego, CA) attached to a Leica M165 FC stereomicroscope (Leica Microsystems, Wetzlar, Germany), and were filmed at a frame rate of 480 000 frames s^−1^ and shutter speed of 940 000 s^−1^. After filming, the wet mass of each individual's whole body, head and mandibles was measured with a UMX2 microbalance (Mettler Toledo).

Strikes were digitized by tracking the x-y coordinates of the distal tip of each mandible throughout the strike using ImageJ v. 1.51r [21]. The angular displacement of the mandible was calculated trigonometrically from the x-y coordinates and the length of the mandible using a custom script. Cumulative displacement was fitted with a quintic spline using the Pspline package in R v. 3.2.2 (R working group), and angular velocity and acceleration were calculated as the first and second derivatives of the curve-fit displacement data. Linear velocity, linear acceleration and power density were also calculated for each strike, and maximum individual performance for each kinematic parameter was used for statistical analysis. Maximum individual performance for each kinematic parameter was calculated from five videos recorded for each individual. We estimated digitization error and tested several numerical differentiation methods (electronic supplementary material).

To estimate the power required for the observed motion, the mandible was modelled as a linear rod, rotating around one end, with a moment of inertia, *I*, of:

$$I=\;\frac{1}{3}mr^{2},$$

where *m* is the mass of the mandible, and *r* is mandible length. The kinetic energy of the mandible, *E*_k_, was calculated by:

$$E_{k}=\;\frac{1}{2}I\omega^{2},$$

where *ω* is the peak angular velocity of the mandible during a strike. The power density of a strike, *P*, was then calculated from:

$$P=\frac{E_{k,\max}}{t_{E,\max}\;M_{\mathrm{add}}},$$

where *E*_k,max_ is the maximum kinetic energy of the mandible, *t*_E,max_ is the time when *E*_k,max_ is at its peak, and *M*_add_ is the mass of the mandible adductor muscle. We used one-half of the whole body mass as a proxy for *M*_add_, which results in an underestimate of power density.

### 2.3. Finite-element analysis and model validation

**Figure 2.**
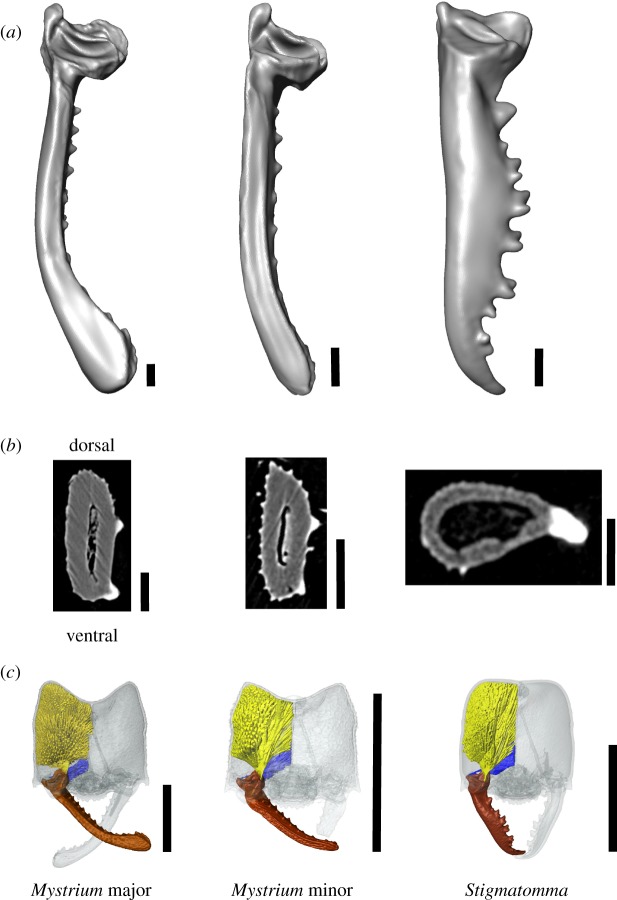
Ant mandibles compared with finite-element analysis. (*a*) Simplified surface models, and (*b*) cross-sections of three ant mandible specimens: the snap-jaw ant *Mystrium camillae* major worker, *M. camillae* minor worker, and the biting jaw ant, *Stigmatomma pallipes*. (*c*) Surface renderings of the head (transparent grey), mandible (brown), mandible adductor muscle (yellow), and mandible abductor (blue) of each specimen. Cross-sections are taken from the mandible bases. Scale bars: (*a*) and (*b*) = 0.1 mm, (*c*) = 1.0 mm.

We used finite-element analysis (FEA) to determine the mechanical consequences of mandible shape variation among power-amplified and non-power-amplified ants. FEA simulates *in silico* how three-dimensional structures respond to applied forces [22–25]. Models for FEA were based on three-dimensional surface renderings of ant mandibles generated by X-ray microtomography (microCT) (figure 2). MicroCT was performed with an Xradia MicroXCT-400 and resulted in image stacks with voxel sizes ranging from 1.68 to 4.24 mm (see electronic supplementary material, table S1).

Mandibles were segmented from the rest of the head and surface meshes were constructed using the software Amira 5.5.0 (FEI, Hillsboro, OR, USA). Extraneous features, such as fine cuticular sculpturing, and mesh imperfections, like holes and non-manifold triangles, were removed or corrected in Geomagic Wrap (3D Systems, Rock Hill, SC, USA). The surface meshes were then imported back into Amira, where mesh quality (triangle aspect ratio and dihedral angle between elements) was confirmed and the number of triangle elements was reduced to approximately 85 000. The resulting STL surface meshes were imported into Strand7 (Strand7 Software Development, Sydney, Australia) where they were used to create four-node tetrahedral volume meshes.

Finite-element analysis was performed in Strand7 using the static linear solver. To mimic the loading phase of the strike, mandibles models were constrained from rigid body motion at the two condyles of the mandible joint and at the mandible tip (figure 1). A force was applied at the point of muscle attachment on the medial base of the mandible to simulate loading by the closer muscle. The magnitude of the applied force, *F*_add_, was estimated from an inverse dynamics analysis of high-speed videos of mandible strikes:

$$F_{\mathrm{add}}=\frac{E_{k,\max}}{\theta d},$$

where *θ* is the angular displacement where *E*_k,max_ is attained and *d* is the in-lever distance between the point of muscle attachment and the centre of mandible rotation (figure 1). The estimated bite force to produce the observed motion was 0.35 N. The cuticle material was modelled to be isotropic and given a Young's modulus of 2.75 GPa and Poisson ratio of 0.3 based on previous measurements of the leaf-cutting ant *Atta* mandibles [26].

**Figure 3.**
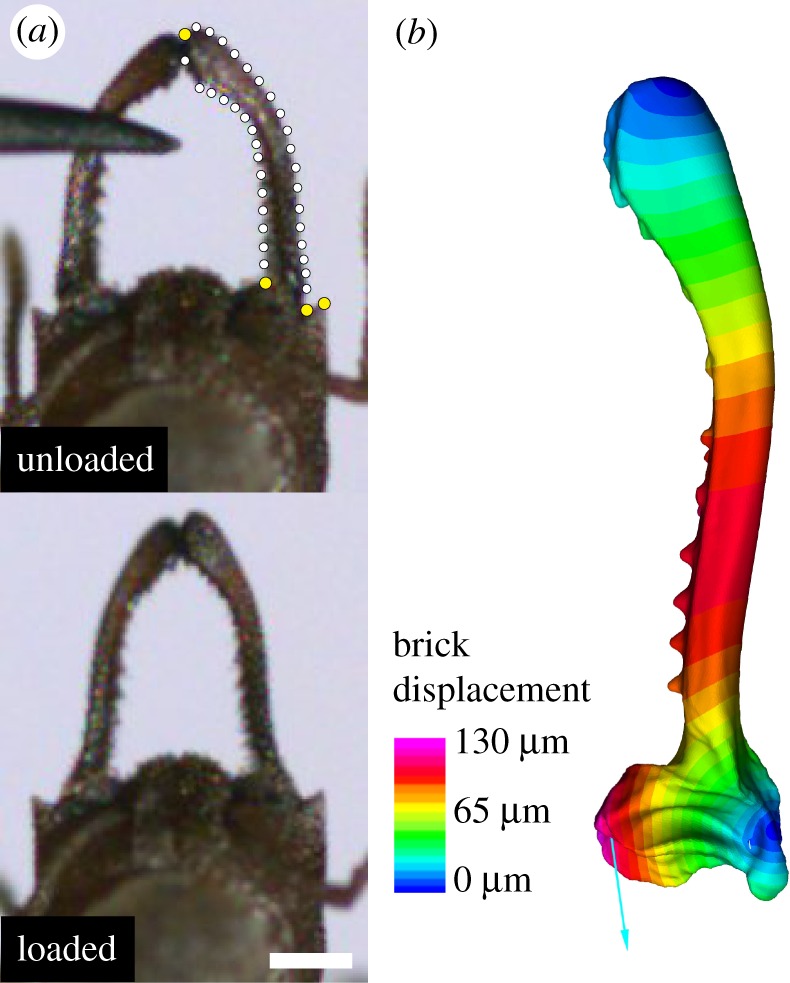
Loading phase and displacement of *Mystrium* mandibles prior to a strike. (*a*) Still images from high-speed video of the loading phase of a strike displaying the mandible in an unloaded (top) and loaded (bottom) state. Dots indicate the fixed landmarks (yellow) and semi-landmarks (white) used in two-dimensional geometric morphometric analysis. (*b*) Loaded finite-element model of striking mandible. Colours correspond to the amount of displacement of each brick element. Scale bar = 0.5 mm.

To confirm that the finite-element model was a realistic representation of *Mystrium* mandible deformation, we compared the simulated mandible displacement in the FEA to the observed displacement of live animals from high-speed strike videos (electronic supplementary material, movie S1). The shape of the striking mandible was quantified with geometric morphometrics from a single frame at the beginning of the loading phase (unloaded) and one from immediately before a strike began (loaded) (figure 3). Thirty-six two-dimensional landmarks were used: four fixed landmarks defining the mandible tip and base, and 32 sliding landmarks that are used to quantify the curvature of the mandible shaft. For the finite-element model, two-dimensional landmarks were placed on images of the undeformed and deformed structure in Strand7. Procrustes superimposition was used to remove the effect of rotation, translation and size using the R package ‘geomorph’, and the resulting aligned Procrustes residuals were subjected to a principle component analysis to examine the morphospace of each sample. A Procrustes ANOVA with permutation procedures was used to assess differences between unloaded and loaded mandibles [27].

### 2.4. Model comparisons

We compared the morphology of *M. camillae* mandibles from the major and minor worker caste to the biting mandibles of the closely related species *Stigmatomma pallipes* [17,19]. *Stigmatomma* ants have a similar ecology to *Mystrium*, foraging underground for centipede prey [28], but do not have power-amplified mandibles (see electronic supplementary material).

To compare mechanical performance, we quantified the von Mises stress and total strain energy resulting from applied loads. Von Mises stress can be used to predict failure and is a good measure of structural strength. For comparison of stress between models, the ratio of force to cross-sectional area was kept constant by scaling all models to the same surface area [22]. Total strain energy is an estimate of work done deforming a structure and is related to the elastic energy stored in the structure (i.e. stiffness). For strain comparisons between models, the ratio of force to volume was kept constant by scaling all models to the same volume. By scaling models in this way and keeping all model parameters uniform, any differences in the estimated stress and strain should be due to variations in model shape.

## 3. Results

### 3.1. The ‘snap-jaw’ mechanism of *Mystrium* ants

In contrast to other ‘trap-jaw’ ants that shut their mandibles from an open position using separate spring, latch and trigger structures, *Mystrium* snap-jaw ants slide their mandibles across each other and combine the spring and latch into the mandible itself [7]. Filming *M. camillae* mandible strikes at 1000 frames s^−1^ allowed characterization of the loading phase and confirmed previous observations of the role the mandible plays as a spring [7] (electronic supplementary material, movie S1). Prior to mandible movement, the tips of both mandibles were pressed against each other and the base of the mandibles bowed inward. This loading phase ranged from 0.66 to 3.7 s and resulted in a narrowing of the mandible gape by approximately 20%. The average mandible base bent inwards by 79.1 µm (s.d. ± 3.1 µm). Mandible movement could not be resolved when filming at this frame rate, indicating that a strike occurs in less than 1 ms.

### 3.2. Snap-jaw kinematics

**Figure 4.**
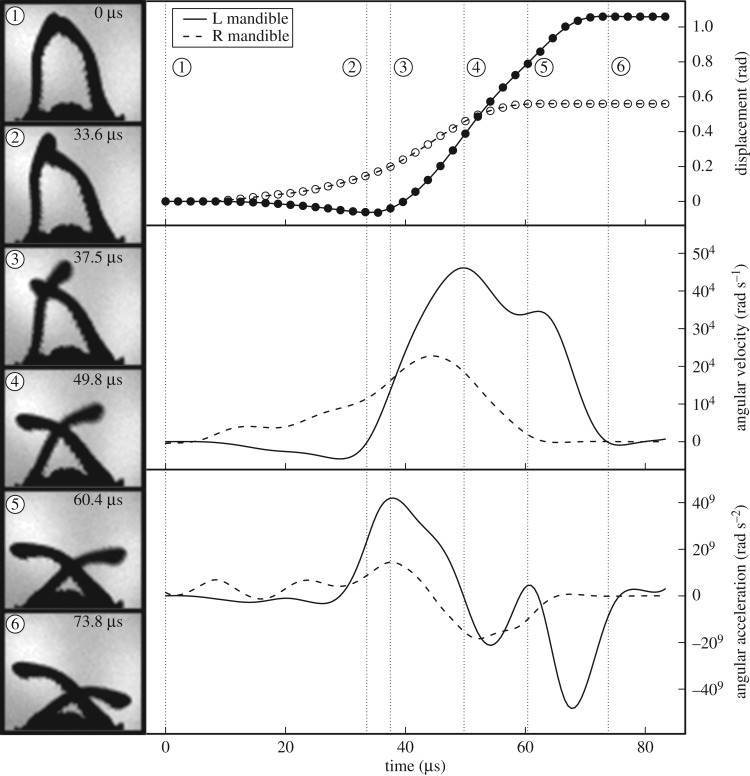
Snap jaw kinematics. *Left*—Still images from a high-speed video of a representative *M. camillae* major worker mandible snap. The time elapsed since the beginning of mandible movement is given. *Right*—Kinematic profiles of the left (solid line) and right (dashed line) mandible derived from a high-speed video of a single mandible snap. Displacement (top panel) of the mandible tips (filled and open circles) was calculated from their *x*-*y* coordinates and smoothed with an interpolated spline. Angular velocity (middle panel) and angular acceleration (bottom panel) were calculated as the first and second derivative, respectively, of the smoothed displacement data. See main text for description of major events during strike and table 1 for means and variance across samples.

Filming strikes at 4.8 × 10^5^ frames s^−1^ permitted visualization of the mandible movement (figure 4 and electronic supplementary material, movie S2). A complete strike occurred in as little 22.9 µs and had an average duration of 50.2 µsec (s.d. ± 22.0 µs). The forward movement of the mandibles was always asynchronous, with one mandible tip beginning its rotation motion 21.9 µsec (s.d. ± 7.4 µsec) before the other. As the first mandible (the ‘loading mandible’) rotated forward, it pushed against the second mandible (the ‘striking mandible’) causing a backwards rotation of 3.5° (s.d. ± 0.89°). The striking mandible began its forward motion by sliding over the loading mandible and accelerating to its peak velocity (average = 7.6 × 10^4^ rad s^−1^, s.d. = ±3.3 × 10^4^) about halfway through its trajectory. After a brief deceleration, the tip of the striking mandible always accelerated a second time as the mandible returned to its normal undeformed shape.

**Table 1.** Morphological and strike performance characteristics of *Mystrium camillae* castes studied. All values are means ± standard deviation. The unit of replication is individual workers (*n* = 5). The minimum or maximum values for each striking mandible performance parameter from 3–5 strikes from each individual were used for summary statistics. Values that are significantly different from each other are listed in italics (*t*-test, *p* < 0.05).

	body mass (mg)	mandible mass (µg)	mandible length (mm)	strike duration (µs)	angular displacement (rad)	peak angular velocity (rad s^−1^)	peak linear velocity (m s^−1^)	peak angular acceleration (rad s^−2^)	peak linear acceleration (m s^−2^)	power density (watt kg^−1^)
major worker	*2.66 ± 0.78*	*85.9 ± 14.4*	*1.56 ± 0.146*	*34.8 ± 4.1*	1.27 ± 0.10	*5.6 × 10^4^ ± 8.7 × 10^3^*	86.9 ± 12.1	*6.0 × 10^9^ ± 2.1 × 10^9^*	*9.3 × 10^6^ ± 3.2 × 10^6^*	*5.0 × 10^6^ ± 1.6 × 10^6^*
minor worker	*0.48 ± 0.06*	*8.5 ± 2.6*	*0.844 ± 0.0595*	*14.6 ± 2.1*	1.19 ± 0.15	*11.1 × 10^4^ ± 18.4 × 10^3^*	93.3 ± 14.5	*17.2 × 10^9^ ± 2.7 × 10^9^*	*14.5 × 10^6^ ± 2.5 × 10^6^*	*2.5 × 10^6^ ± 1.1 × 10^6^*

The asynchrony of the two mandibles resulted in significant kinematic differences between them (table 1; electronic supplementary material, figure S1). The striking mandible rotated about twice the distance as the loading mandible, and in two-thirds the time (paired *t*-test: distance, *t* = −29.05, *p* < 0.001; duration, *t* = 12.19, *p* < 0.001). The striking mandible also had significantly higher peak velocity and acceleration (paired *t*-test: angular velocity, *t* = −11.51, *p* < 0.001; angular acceleration, *t* = −9.57, *p* < 0.001). The power density of both mandibles exceeds the limits of direct muscle contraction, but the striking mandible is over seven times more powerful than the loading mandible (paired *t*-test: *t* = −12.6, *p* < 0.001).

We found significant differences in strike kinematics between castes of *M. camillae* (table 1). The striking mandible of smaller minor workers had significantly shorter strike duration, and higher angular velocity and acceleration than larger major workers (Welch two sample *t*-test: duration, *t* = 9.8, *p* < 0.001; angular velocity, *t* = −6.0, *p* < 0.001; angular acceleration, *t* = −7.4, *p* < 0.001). There was no difference between castes in the distance travelled by the striking mandible (*t* = 0.95, *p* = 0.37), which, due to differences in mandible length, resulted in no difference in the linear peak velocity of the striking mandible (*t* = −0.75, *p* = 0.47). Major workers had strikes that were twice as powerful as minor workers (*t* = 2.88, *p* = 0.02), reflecting the larger mass of their mandibles.

### 3.3. Finite-element model validation

**Figure 5.**
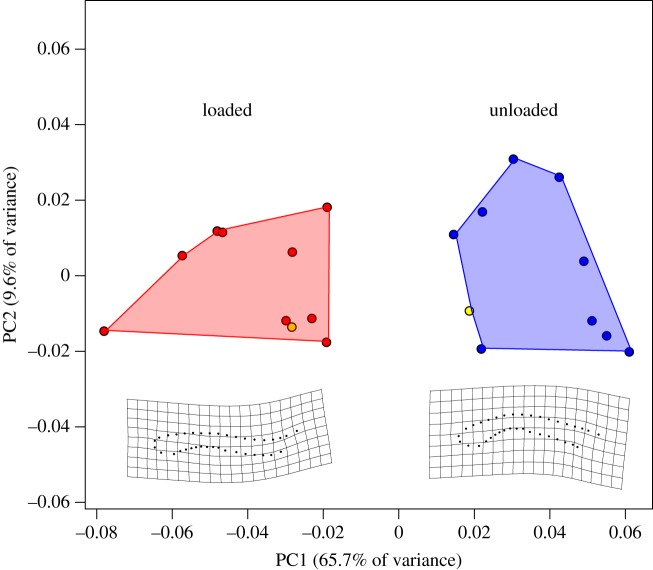
Principle component analysis of mandible shape during loading. Procrustes-aligned two-dimensional landmarks clustered in two different groups in morphospace: unloaded mandibles at the beginning of a strike that had not yet deformed (blue), and loaded mandibles immediately prior to the strike (red). The shapes of unloaded and loaded mandibles from the finite-element models are displayed in yellow. Thin plate splines (below) display the minimum and maximum shapes of the first principle component which accounted for 65.7% of the total shape variance.

Our finite-element model roughly approximated the displacement of striking mandibles of live animals. In the finite-element analysis, the basal portion of the mandible shaft was displaced approximately 90 µm, which is the same order of magnitude of displacement that was observed in videos (approximately 70 µm) (figure 3). The two-dimensional landmarks displayed the same pattern of mandible displacement in both the FE model and the high-speed videos, with the basal portion of the mandible shaft bending medially (figure 5). There was a significant difference in mandible shape between the unloaded and loaded mandibles (Procrustes ANOVA: *R*^2^ = 0.547, *F* = 21.7, *p* < 0.001), and the shapes of the two FE specimens clustered with their respective groups. These results confirm that the finite-element model is a similar approximation of the mechanical deformation that is occurring in the live animal.

### 3.4. Model comparisons

Although they share morphological features, microCT revealed several differences between the snap-jaw mandibles of *Mystrium camillae* and the biting mandibles of *Stigmatomma pallipes* (figure 2; electronic supplementary material, movie S3). As is common throughout the subfamily, both species have long and linear mandibles that are widely set on the head. The mandibles of both species are also elliptical in cross-section, but in different orientations. The basal portion of the mandible shaft of *Mystrium* mandibles are flattened laterally, whereas *Stigmatomma* are flattened dorsoventrally. Additionally, the inner masticatory margin of *Stigmatomma* mandibles are lined with well-developed teeth, including a sharp apical tooth. *Mystrium* mandibles, in contrast, have relatively reduced teeth and denticles, and the apical tooth has been rotated ventrally, making the apical portion of the mandible club-like [17].

**Figure 6.**
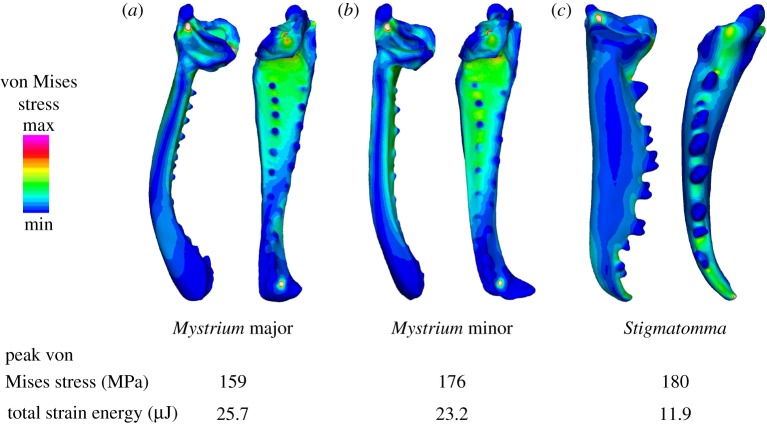
Comparison of snap-jaw and biting mandible morphology. Finite-element models for (*a*) *M. camillae* major worker, (*b*) *M. camillae* minor worker, and (*c*) *S. pallipes* worker. All models were scaled to the same surface area to compare stress. Dorsal (left) and medial (right) views of each mandible displays the distribution of von Mises stresses. Peak von Mises stress and total strain energy are listed below. Contour plots are scaled to von Mises stress between 0 and 250 MPa.

The distribution of von Mises stress, an estimate of structural strength, calculated from the finite-element analysis on each species mandible during muscle loading is displayed in figure 6. Model elements around the constrained nodes and force load had exceptionally high stresses, probably artefacts due to applying constraints and loads on single elements [29]. Consequently, we limited the comparison of maximum stresses to the shaft of each mandible. In all three specimens, the region of highest stress was found on the inner and outer margins of the mandible, with peak stresses near the base. There was little difference in the maximum von Mises stress among the three specimens, indicating that they were all equally likely to fail. The major worker of *M. camillae* had the lowest stress (159 MPa), but it was only 11% lower than *Stigmatomma* and the minor worker of *M. camillae* (174 and 176 MPa, respectively). In contrast, there were large differences between species in the total strain energy, which is related to flexural stiffness. Both castes of *Mystrium* had approximately twice the total strain energy (25.7 and 23.2 µJ) of *Stigmatomma* (11.9 µJ), and thus are more likely to deform when loaded in this way.

## 4. Discussion

With peak linear velocities around 90 m s^−1^ and accelerations above 10^6^ × *g*, *M. camillae* mandible snaps are the fastest power-amplified movement to be recorded in arthropods, and among the fastest biological movements generally. The duration of the snap movement is one thousand times shorter than the snap of a human finger and five thousand times shorter than the blink of an eye [30,31]. The power-amplified mandible strikes of other trap-jaw ants, such as the genera *Odontomachus* and *Myrmoteras*, take three to sixty times longer than the snaps of *Mystrium* [20,32,33] and achieve peak velocities that are ten to twenty times slower than *Mystrium*. Snap-jaw ant mandibles exceed the velocities and accelerations of other arthropod power-amplified movements, including trap-jaw spider chelicerae (8.5 m s^−1^, 10^3^ × *g*) [34], mantis shrimp dactyls (30 m s^−1^, 10^4^ × *g*) [35], and froghopper jumps (4.7 m s^−1^, 10^2^ × *g*) [36], and have 10–75 times the power density of these movements. The only other animal with comparable performance to *Mystrium* ants are termites in the genus *Termes*, whose soldier caste have a similar mandible snapping mechanism that occurs in less than 0.025 ms and achieves velocities of 67 m s^−1^ [8].

The high accelerations of *Mystrium* strikes probably result in high impact forces necessary for predatory or defensive behaviours. Other predatory arthropods with similar power-amplified appendages produce forces that are many times greater than their body weights. The dactyl of mantis shrimp, for example, reach peak impact forces up to 1500 N to crack mollusc shells [37]. Similarly, the mandibles of trap-jaw ants in the genus *Odontomachus* generate impact forces up to 500 times their body weight to stun prey or to power defensive escape jumps [32]. Although we were unable to directly measure the strike forces of *Mystrium* strikes, it is not unreasonable to predict they are also producing disproportionately high impact forces to stun or kill prey.

It is notable that the two fastest arthropod appendage movements (*Mystrium* and *Termes*) share the same snapping mechanism for power amplification. One explanation for this is that both snap-jaw ants and termites are accelerating their mandibles over a much shorter distance than other power-amplified animals, and their performance reflects trade-offs in power-amplified systems between appendage velocity, displacement and motor force [6]. The high performance of snap jaws also might be related to the efficiency of having the mandible behaving as both spring and projectile. Most other power-amplified appendages have distinct structures that act as motor, spring, latch and tool [3]. By combining spring and tool in the same appendage, the snapping mechanism may transfer energy more efficiently between the muscle and the mandible. A broad comparative study including other snapping mechanisms, such as the ant genus *Plectroctena* [9] and the termite genus *Capritermes* [10], would confirm that snap jaws are a qualitatively higher performing power-amplification mechanism and also contribute to an explanation of how this arrangement of components might lead to the high performance of snap jaws.

The intraspecific scaling of snap-jaw performance mirrors that found among trap-jaw ants and other power-amplified appendages in general. Strike performance in the genus *Odontomachus* scales negatively with body size, with smaller ants having faster strikes and higher accelerations [33]. Acceleration generally scales negatively with body size across most animal systems [4,6]. Major *Mystrium* workers, however, have more powerful strikes than minor workers because of their more massive mandibles. This difference in power performance may reflect a caste-based division of labour: larger workers use their more powerful mandibles for defence and foraging on predatory arthropods, like centipedes, whereas smaller workers and ergatoid queens perform tasks within the nest such as brood care [11,14,15].

Our FEA analysis found no difference in peak von Mises stress between snap jaws and biting jaws, but did find higher total strain energy of snap jaws. Total strain energy is related to flexural stiffness, and our results suggest that snap jaws are less stiff than biting jaws. This is consistent with the *Mystrium* mandible's function as a spring, storing elastic energy that is required to power the mandible strike. The difference in stiffness between snap-jaw and biting mandibles is probably due, in large part, to differences in cross-sectional geometry. The cross-sectional shape of a structure defines the second moment of area, a key component of flexural stiffness [38]. In contrast to the non-power-amplified mandibles of *Stigmatomma* (and, indeed, most ant mandibles), the base of *Mystrium* mandibles are flattened laterally (figure 2). This elliptical shape concentrates mass near the neutral bending axis of mandible, reducing the second moment of area and the stiffness of *Mystrium* snapping mandibles. In contrast, biting mandibles, like those of *Stigmatomma*, concentrate mass further from the neutral bending axis in order to resist deformation. Together, these data reflect how the morphology of *Mystrium* mandibles correlate with its mechanical and kinematic performance, and are specialized for energy storage and power amplification.

Our FE model closely matched the displacements observed in live animals, but elastic strain energy used to power the mandible strike may not be limited to the mandibles. Other trap-jaw ants also store elastic energy in the cuticle of their heads, apodemes, and probably in the muscles themselves. Future work should attempt to fully account for the energetics powering the *Mystrium* strike, including empirically quantifying the strain energy stored in the mandible cuticle and other head structures.

The results from our morphological examination and FEA is another example of how relatively small changes in mandible shape could lead to the evolution of power amplification [39,40]. This transition may have been particularly easy in the ant subfamily Amblyoponinae given mandible traits that are common to species in the subfamily. Widely set, elongate mandibles are necessary for a snapping mechanism where one mandible crosses over the other, and this trait has evolved multiple times in the subfamily [17,41]. Obviously, a shape change of the mandible base was not the only alteration required for the evolution of power amplification. It was probably accompanied by an increase in mandible adductor volume to power the strike and the neural mechanisms to control the strike's loading and release. More research on the basic biology of these cryptic ants is needed to determine why a ‘snap-jaw’ mechanism has evolved in *Mystrium*. *Mystrium camillae* are at the top of ant food webs in Southeast Asia, probably foraging exclusively on predatory arthropods [42]. The foraging and nesting habits of *Mystrium* are also restricted to confined tunnels in logs and in the soil [11], and this may favour this type of amplification system where the ant cannot open its jaws widely, as seen in trap-jaw ants, which largely forage in open spaces (e.g. the surface).

Our FEA results provide an idea of the kind of morphological changes required to evolve snap jaws from biting jaws, but the snap jaws of *Mystrium* and other insects may be an ideal system for studying spring and latch dynamics of power-amplified systems more generally. The spring and latch of snap jaws are easily observable and more amenable to manipulative experiments than other insect power amplification systems, where components are often hidden within the exoskeleton. Considering there are at least 14 species of *Mystrium*, there is also tremendous opportunity to measure the relationship between changes in spring morphology or material properties and performance. Future comparative studies that include other independent lineages of snap jaws can take advantage of the morphological diversity to better understand the complex interactions that underpin high-speed animal performance.

## Supplementary Material

Supplementary Methods

## Supplementary Material

Movie S1. High-speed video of snap-jaw strike loading phase.

## Supplementary Material

Movie S2. High-speed video of a snap-jaw strike.

## Supplementary Material

Movie S3. Surface renderings of heads and mandibles of the three specimens used for FEA.

## Data accessibility

Morphological data (microCT image stacks and surface volumes) are available through MorphoSource (morphosource.org, project ID: P539). *x*-*y* coordinate data of strikes, derived kinematic characteristics, and R scripts for statistical analyses are available from Dryad Digital Repository at http://dx.doi.org/10.5061/dryad.4863215 [43].
