# Supplementary material for: Snap-jaw morphology is specialized for high-speed power amplification in the Dracula ant, Mystrium camillae
Source: R Soc Open Sci. 2018 Dec 12;5(12):181447. doi: 10.1098/rsos.181447 (PMC6304126; doi:10.1098/rsos.181447)
Supplement: Supplementary Methods [file rsos181447supp1.docx]

**Snap-jaw morphology is optimized for high-speed power amplification in the Dracula ant, *Mystrium camillae***

Fredrick J. Larabee, Adrian A. Smith, Andrew V. Suarez

SUPPLEMENTAL MATERIAL

**Digitization Error of Kinematic Analysis**

Digitization error was assessed by tracking mandible strikes from three videos five times each. The variance in mandible tip tracking was quantified by calculating the centroid for the x-y coordinates of the mandible tip in each frame. The mean centroid size across all frames was 1.166 pixels (s.d. ± 0.5698), with no difference in centroid size between the three strikes (one-way ANOVA: *F*=1.4493, *P*=0.2513). To measure the effect of this digitization variance on the estimation of kinematic parameters, we estimated the coefficient of variation (CV) in cumulative displacement, maximum angular velocity, and angular velocity for each strike. Variation in kinematic parameters increased with derivation of displacement data, with the mean CV for cumulative displacement being lowest and acceleration being the highest (2.61 % and 19.9 %, respectively).

The quintic spline used to smooth the displacement data was compared with other differentiation methods to assess its appropriateness. Average velocity and acceleration were calculated from the difference between frames in mandible displacement and velocity, respectively, and compared with parameters derived from the smoothed positional data (Fig. S1). Maximum velocity and acceleration calculated from the smoothed positional data were both slightly lower than when calculated from between frame differences (paired Wilcoxon signed rank test, velocity: V=114, P=0.000854; acceleration, V=120, P < 0.00001), indicating that our kinematic estimates are conservative estimates of mandible performance.

**Figure S1. Comparison of differentiation methods on *Mystrium* strike kinematic data.** X-y coordinate data was smoothed with a quintic spline (solid line) and then first and second derivatives were used to estimate velocity and acceleration, respectively. For comparison, velocity and acceleration was estimated by taking the difference between frames in position and velocity, respectively (dotted line).


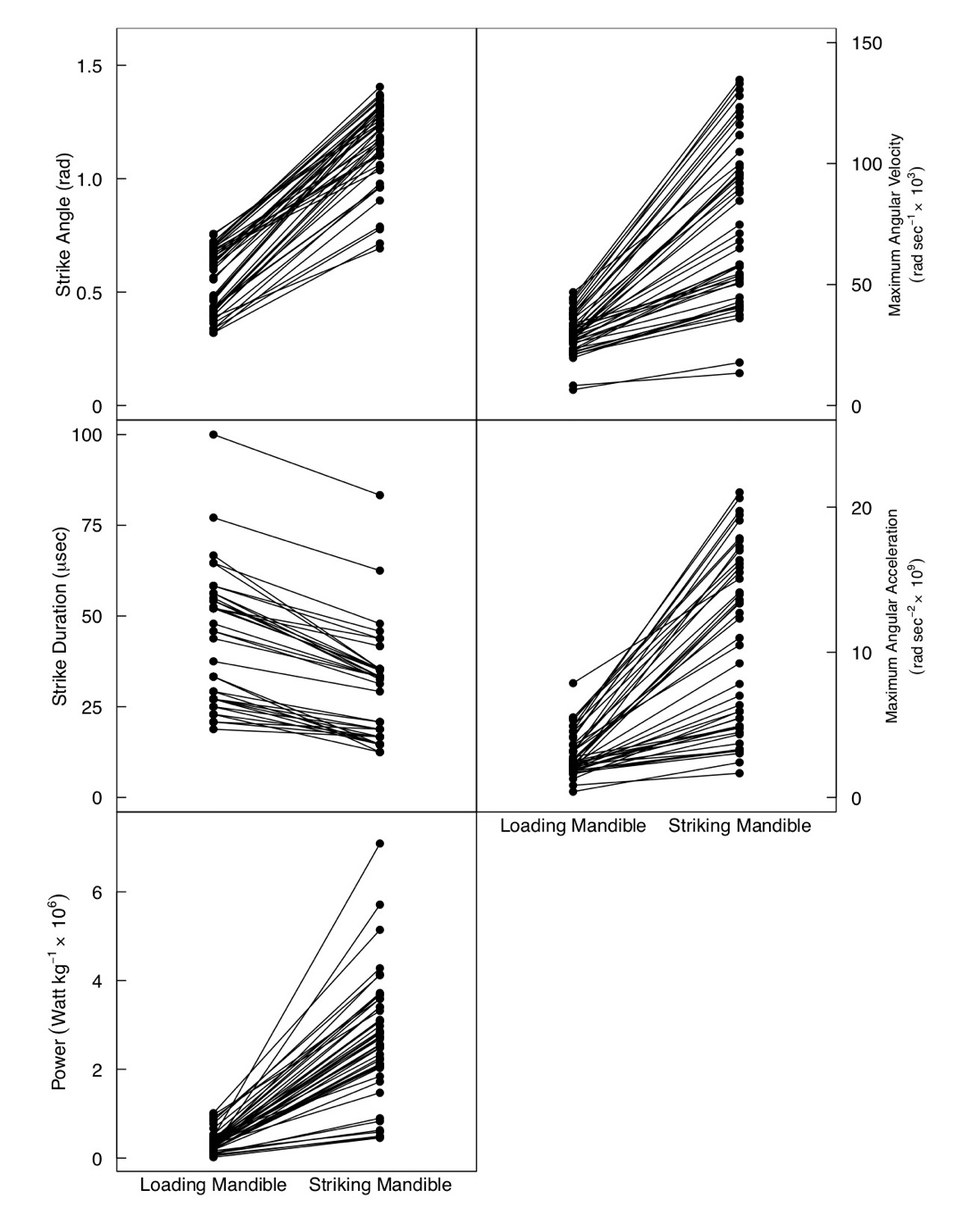


**Figure S2. Asymmetry in mandible performance.** Paired comparisons of loading mandible and striking mandible performance. All comparisons were significantly different (paired t-test, P<0.05).

***Stigmatomma* Kinematics**

For *Stigmatomma* mandible closures, six bites from two ants were filmed at 4900 s^-1^ with a Phantom Miro LC320s high speed camera (Vision Research Inc., Wayne, NJ) through a Vision Optics Laowa 60mm f/2.8 2X Ultra-Macro Lens. In contrast to the snapping mandibles of *Mystrium*, the non-snapping mandibles of *Stigmatomma* were very slow. From measurements of six bites from two ants, their mandibles closed in as little as 31.2 ms. They achieved average peak angular velocities of 67.1 rad s^-1^ (SD ± 13.1 rad s^-1^) and average angular accelerations of 1.9 x 10^4^ rad s^-2^ (SD ± 4.9 x 10^3^ rad s^-2^). The mandible and muscle mass of *Stigmatomma* was not measured, but it is unlikely that the power density of their bites exceeds the limits of their mandible adductor muscle.

**Table S1.** Scanning parameters for X-ray tomography.

| **Specimen** | **Voucher Number** | **Voltage**  **(kV)** | **Current (mA)** | **Exposure**  **(s)** | **Pixel Size (mm)** | **Head Volume**  **(10^-1^ mm^3^)** | **Adductor**  **(% Vol.)** | **Abductor**  **(% Vol.)** | **Mandible**  **(% Vol.)** | |
| --- | --- | --- | --- | --- | --- | --- | --- | --- | --- | --- |
| ***Mystrium camillae* major** | USNMENT01124409 | 20 | 100 | 25 | 3.21 | 18.53 | 54.8 | 2.3 | | 3.2 |
| ***Mystrium camillae* minor** | USNMENT01124410 | 25 | 100 | 20 | 1.677 | 4.26 | 35.7 | 1.1 | | 2.2 |
| ***Stigmatomma pallipes*** | USNMENT01124411 | 25 | 200 | 7 | 3.358 | 7.84 | 43.1 | 1.9 | | 2.7 |

**Supplementary Movie Legends**

**Supplementary Movie S1. High-speed video of snap-jaw strike loading phase.** Representative video used for visualization of the loading phase and for FEA validation**.** Filmed at 1000 frames s^-1^.

**Supplementary Movie S2. High-speed video of a snap-jaw strike.** Representative video of a jaw strike of a major worker of *Mystrium camillae*. This strike was used to produce figure 2. Filmed at 480,000 frames s^-1^.

**Supplementary Movie S3. Surface renderings of heads and mandibles of the three specimens used for FEA.** Mandibles (top) were defeatured and smoothed for finite element analysis. Heads (bottom) were used to visualize mandible muscle morphology and to determine points of attachment for muscle. Mandible adductor muscle (yellow), mandible abductor (blue), and mandible (brown) are displayed. Models are not to scale.
